# Azole Combinations and Multi-Targeting Drugs That Synergistically Inhibit *Candidozyma auris*

**DOI:** 10.3390/jof10100698

**Published:** 2024-10-07

**Authors:** Stephanie Toepfer, Mikhail V. Keniya, Michaela Lackner, Brian C. Monk

**Affiliations:** 1 Sir John Walsh Research Institute, University of Otago, Dunedin 9016, New Zealand; stephanie.toepfer@i-med.ac.at; 2 Institute of Hygiene and Medical Microbiology, Medical University Innsbruck, 6020 Innsbruck, Austria; michaela.lackner@i-med.ac.at; 3 Center for Discovery and Innovation, Hackensack Meridian Health, Nutley, NJ 07110, USA; mikhail.keniya@hmh-cdi.org

**Keywords:** combination therapy, antifungal, *Candida* spp., *Candida auris*, *Candidozyma auris*, synergy, alternative therapy, yeast

## Abstract

Limited antifungal treatment options and drug resistance require innovative approaches to effectively combat fungal infections. Combination therapy is a promising strategy that addresses these pressing issues by concurrently targeting multiple cellular sites. The drug targets usually selected for combination therapy are from different cellular pathways with the goals of increasing treatment options and reducing development of resistance. However, some circumstances can prevent the implementation of combination therapy in clinical practice. These could include the increased risk of adverse effects, drug interactions, and even the promotion of drug resistance. Furthermore, robust clinical evidence supporting the superiority of combination therapy over monotherapy is limited and underscores the need for further research. Despite these challenges, synergies detected with different antifungal classes, such as the azoles and echinocandins, suggest that treatment strategies can be optimized by better understanding the underlying mechanisms. This review provides an overview of multi-targeting combination strategies with a primary focus on *Candidozyma auris* infections.

## 1. Introduction

Fungal pathogens cause more than two billion significant infections of humans each year and about 1.6 million attributable deaths. The largest group of species within the diverse kingdom of fungi is the Ascomycota. Particularly important members of the Ascomycota from the clinical viewpoint are *Candida* spp., which feature strongly in the list of fungal pathogens designed by the World Health Organization (WHO) to guide research, therapeutic development, and public health action [1]. 

Some *Candida* spp. are commensals of the human body. As part of a healthy microbiome, they can colonize the skin [2], gastrointestinal tract [3], and oral cavity [4]. These *Candida* representatives can also be opportunistic pathogens of immunocompromised or immunologically impaired people [5,6] and are among the most common human fungal pathogens [7], causing diseases described as “candidiasis”. Invasive candidiasis is a growing concern often linked to the use of indwelling medical devices such as ventilators, urinary catheters, and central venous catheters [8,9,10] with a high mortality rate (between 10% and 47%) [11]. 

Among candidiasis causing pathogens, *Candida albicans* is the pathogen most frequently isolated in the clinic, followed in order of incidence by non-*albicans* species *Nakaseomyces glabratus* (=*Candida glabrata*), *Candida parapsilosis*, *Candida tropicalis*, and *Pichia kudriavzevii* (=*Candida krusei*) [8,10,12]. *Candidozyma auris* (=*Candida auris*) has emerged recently as a threat responsible for nosocomial outbreaks across the globe [13,14] and has been recognized as major a global risk by both the CDC and the WHO [1,15,16,17]. 

Rapid diagnosis with early response, including source elimination (e.g., removal of infected catheters) and appropriate antifungal therapy, are key to the effective management of invasive candidiasis [18,19]. Antifungal monotherapy of *Candida* infections remains the recommended standard [19]. Management guidelines have yet to include combination therapy, except in some specific circumstances, e.g., Flucytosine can be combined with Amphotericin B (AMB) to treat *Candida* endocarditis [19]. 

Numerous studies have identified two-compound combinations that act synergistically against multidrug resistant microbial pathogens [20,21,22,23,24]. However, combination therapy might only be considered for the salvage of patients who are refractory to antifungal monotherapy. The use of lower effective antifungal concentrations in synergistic multi-drug combinations has potential benefits that include reduced host toxicity, and shorter treatment regimens [25]. Better efficacy due to the involvement of multiple targets theoretically reduces the risk of resistance development, and potentially increases the economic life span of antifungal drugs [26], e.g., by repurposing existing drugs [27]. However, only a few antifungal combinations have been tested successfully in clinical trials (reviewed in Fioriti et al. [28]). 

The following sections of this review will briefly describe the major antifungal drug classes used in the clinic against the genus *Candida* and related genera, as well as a selection of compounds in the antifungal discovery and development pipeline with potential to contribute to combination therapies. 

## 2. Antifungal Agents 

**Figure 1:**
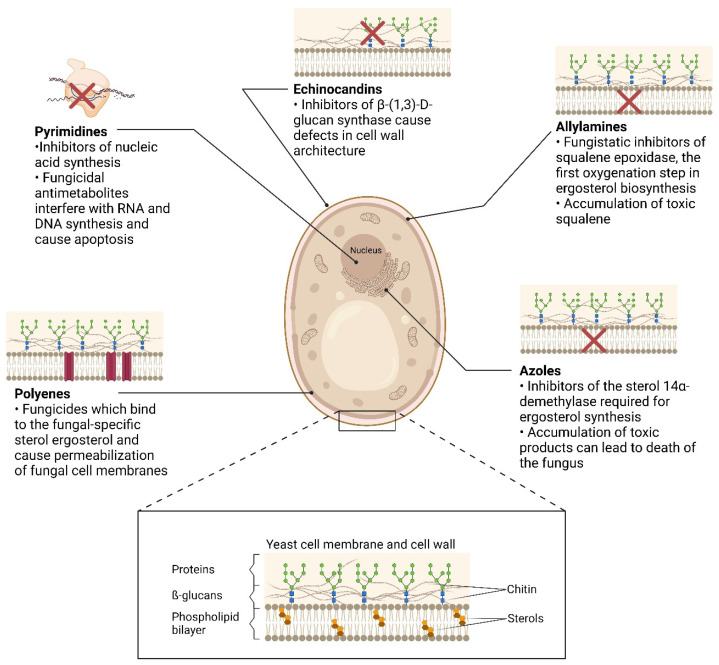
Mode of action of the five main antifungal drug classes used to treat Candida infection [40,41,42,43,44,45]. Created in BioRender. Toepfer, S. (2023) BioRender.com/e45w002.

The chemistry and site of action of current antifungal drugs divides them into five major classes: echinocandins, polyenes, pyrimidines, azoles, and allylamines (Figure 1) [29]. Of these, only the first four are used to treat invasive candidiasis. The allylamines, such as terbinafine, are instead used to treat superficial infections caused by dermatophytes [29]. Depending on their pharmacokinetic effects on the fungus being treated, existing antimycotic drugs are either fungistatic agents that inhibit fungal growth and/or fungicides that kill fungi [30]. For example, the echinocandins exhibit species-dependent fungicidal or fungistatic activity [31]. Azole drugs are, in general, considered fungistatic against human pathogens, but can be rendered fungicidal if delivered to their target in appropriate concentrations by circumventing their susceptibility to drug efflux [32,33].

The increasing incidence of invasive fungal infections (IFIs) [34], poor therapeutic outcomes exacerbated by side effects, and the growing impact on therapy by antifungal resistance [34,35,36] are major concerns. Furthermore, as both fungi and mammalian cells are eukaryotes, their shared genetic, molecular, and cellular properties make it difficult to identify new and druggable fungal-specific targets [37,38]. For example, Liu et al. showed that out of 240 conserved fungal genes, 115 share high similarity (>50%) with human homologs [39]. An additional limitation is that some fungi are innately pan-resistant to the limited range of antifungals in clinical use or can successively acquire resistance to individual antifungals. These problems highlight the importance and difficulty in developing new drugs, such as antifungal combinations, that are active and specific against fungal infection. 

### 2.1. Echinocandins

Echinocandins are relatively expensive, semisynthetic molecules that inhibit β-(1,3)-d-glucan synthase (GS) and block the biosynthesis of the essential β-(1,3)-d-glucan component of the fungal cell wall, a polymer that is not found in mammals [46,47,48]. They target the catalytic subunit of GS which is encoded by the genes *FKS1*, *FKS2*, or *FKS3* in *Saccharomyces cerevisiae*, *FKS1* and *FKS2* in *N. glabratus* and *GSC1* (Glucan Synthase Catalytic Subunit 1, known as *FKS1*), *GSL1* (Glucan Synthase Like 1; known as *FKS3*), and *GSL2* (known as *FKS2*) in *C. albicans* [49,50].

The inhibition of β-(1,3)-d-glucan synthesis weakens the cell wall, leading to osmotic instability and cell death [40]. However, treatment with echinocandins induces cellular stress that can activate adaptive protective mechanisms, such as an upregulation of chitin synthesis that increases the tolerance of echinocandins [51,52]. Representative examples of echinocandin drugs include Micafungin (MFG), Caspofungin (CAS), and Anidulafungin (ANA). Resistance to echinocandins involves target modifications in hotspot regions of GS (regions where resistance mutations most commonly occur) and does not appear to involve drug efflux (i.e., MFG is not transported by efflux pumps) [53]. Echinocandin resistance previously occurred infrequently [54]; however, resistance has recently been detected in up to 8% of treatments [55]. The echinocandins have become the frontline treatment recommended for invasive *Candida* infections in the hospitals where they can be afforded [11].

### 2.2. Polyenes

Polyenes such as AMB are complex macrolide antibiotics [56]. Several hypotheses have been proposed to describe their mode of action. The previously widely accepted model involved the formation of ion-permeable transmembrane channels that lead to membrane permeability and fungal death [41,42]. A dose dependence between polyene concentration and transmembrane channel diameter has been demonstrated [57]. In contrast, the more recent “sterol sponge” theory hypothesizes that AMB extracts ergosterol from the lipid bilayer by forming extramembranous aggregates [58,59]. 

AMB exhibits excellent broad-spectrum activity against many fungal pathogens. However, its dose-dependent host toxicity is a limiting factor [60] that has been partially addressed in various ways, including with liposomal formulations [61,62,63]. Some *Clavispora lusitaniae* isolates exhibit innate resistance to AMB often linked to very low ergosterol content in their membranes [64,65]. In contrast, high ergosterol content in recombinant *S. cerevisiae* strains can reduce susceptibility in a dose-dependent manner to AMB [66].

### 2.3. Allylamines 

Allylamines, such as Terbinafine (TBF), are inhibitors of squalene epoxidase, an early step in ergosterol biosynthesis [43]. The accumulation of toxic squalene is associated with a reduction in cellular ergosterol content, and high amounts of squalene interfere with fungal cell membrane function and cell wall synthesis [67]. Fungal cells then grow poorly because of inefficient nutrient uptake. TBF accumulates in skin and hair more than in blood plasma, explaining why it and other allylamines are particularly effective against dermatophytes [68]. Allylamines can be administered orally or topically and are the first-choice treatment for dermatophyte (skin and nail) infections [69,70], but their potential for drug interactions requires monitoring in some patients both before and during treatment [71,72]. The incidence of resistance to allylamines such as TBF is generally considered to be low; however, cross-resistance has been detected in *S. cerevisiae* strains overexpressing *MDR1* or *CDR1* [73]. 

### 2.4. Pyrimidines

Pyrimidines such as Flucytosine are used to treat infections caused by *Cryptococcus* and *Candida* spp. [74]. Flucytosine is metabolized by fungal cells to 5-fluorouracil which subsequently integrates into the fungal RNA, disrupting RNA synthesis [44]. In addition, the inhibition of thymidylate synthase by 5-fluorouracil contributes to DNA damage that promotes cell death [75]. Flucytosine monotherapy frequently results in acquired resistance [76], and combination with AMB is used to reduce its incidence [77,78,79]. Side effects such as bone marrow toxicity or liver and kidney damage are frequently associated with long-term treatment or the use of high-dose Flucytosine [76]. Due to excellent penetration into body compartments, including the central nervous system, Flucytosine is often used to treat cryptococcal or *Candida* meningitis [80,81]. 

### 2.5. Azoles

Compared with the semi-synthetic echinocandins, the manufacture of azole drugs is inexpensive, and they continue to play important roles in the treatment of IFIs. These drugs have aromatic elements including a heterocyclic ring containing at least one nitrogen atom capable of binding to the iron of heme [82]. The two major subgroups of the azole antifungal drugs are the imidazoles and 1,2,4-triazoles [83]. A third subgroup, the tetrazoles, entered the clinic more recently [84]. 

Azoles are fungistatic drugs used widely to treat infections caused by *Candida* spp. [19,85]. In general, azole drugs disrupt the sterol biosynthetic pathway that produces the fungal-specific sterol ergosterol [45,86]. By limiting the ergosterol content of cell membranes, and by causing the accumulation of toxic intermediates such as 14α-methyl sterols [45], azole antifungals reduce cell membrane stability and the activity of key membrane bound enzymes [45]. They target sterol 14α-demethylase (also known as Erg11 or Cyp51 in yeast and CYP51 in molds) members of the CYP51 group in the cytochrome P450 superfamily of enzymes encoded by *ERG11*/*CYP51* genes. First described in the model yeast *S. cerevisiae*, the Erg11 enzyme catalyzes the removal of the 14α-methyl group from lanosterol [87]. In *Candida* species, both lanosterol and eburicol serve as major substrates of the enzyme, which therefore should be described as a sterol 14α-demethylase [88]. The active site of Erg11/Cyp51/CYP51 contains a heme cofactor where the sixth position of the iron can be occupied by azole drugs, via a nitrogen of an imidazole, triazole, or tetrazole ring [89,90,91].

Compared with imidazoles the triazoles were safer to use and showed a higher selectively and, most importantly, an even lower affinity to human Cyp51s than fungal Cyp51 [82]. The long-tailed triazole Posaconazole (POS) was developed from ITC in a successful effort to increase spectrum and potency and reduce side effects. The triazoles Isavuconazole (ISA) and Ravuconazole (RVC) have mid-length tails. ISA is usually delivered as the prodrug Isavuconazonium sulfate while the investigational drug RVC can be delivered as the prodrug Fosravuconazole. Compared with FLC or VRC, POS and ISA show extended spectrum activity and improved potency against *Candida* spp. and molds such as *Aspergillus* spp. [92,93,94]. ISA differs from conventional azole drugs by having a 2,5-difluorophenol head group instead of a 2,4-fluorophenol head group. RVC has yet to be approved for clinical use [95] despite being highly effective in vitro against *Candida* spp. and *Aspergillus* spp. [96,97]. Its prodrug Fosravuconazole L-lysine ethanolate (F-RVC) has been successfully used in clinical trials in Japan against onychomycosis [98,99] and a clinical trial in Sudan against the dermatophyte disease eumycetoma will soon be reported [100].

### 2.6. The Antifungal Pipeline

The incidence of therapeutic failure due to drug interactions and the development of drug resistance have encouraged the discovery of numerous novel antifungals, some of which are in development. There are several excellent reviews that discuss the discovery, development, clinical trial phases, and efficacy of such antifungals [101,102,103,104,105]. Table 1 shows a selection of promising drug candidates active against *Candida* species.

## 3. Antifungal Combination Therapy

Microbiological and consequent clinical resistance are an increasingly important consideration for medical mycology. Microbiological resistance can be either intrinsic (primary) or acquired (secondary) while clinical resistance is defined as a fungal infection that persists despite the recommended antifungal drug therapy [124]. Intrinsic resistance occurs naturally in fungal species without prior exposure to antifungal drugs. Acquired resistance is found in formerly susceptible strains after exposure to antifungal treatment [125]. Microbiological resistance in vitro is defined according to standard susceptibility testing guidelines. The European Committee on Antimicrobial Susceptibility Testing (EUCAST) and the Clinical & Laboratory Standards Institute (CLSI) have separately created guidelines to identify the minimal inhibitory concentrations (MICs) for yeasts and mold strains to antifungal drugs and have also established clinical breakpoints to interpret MIC data. 

As options for antifungal treatment are often limited, antifungal resistance can result in severe health problems and an increased likelihood of unfavorable treatment outcomes. Among *Candida* clinical isolates in the U.S., 7% of all disseminated infections tested at the CDC were resistant to FLC [126]. Together with an increasing incidence of azole resistant clinical isolates, resistance to the echinocandin drugs has been documented, especially in *N. glabratus* [48,127]. Even so, echinocandins remain the first choice for treating candidemia caused by *N. glabratus*. Reduced susceptibility to echinocandins among strains with intrinsic resistance to FLC severely limits treatment options and greatly increases the likelihood of poor clinical outcomes [128]. In addition to *N. glabratus*, echinocandin resistance has been observed in many *Candida* genera including *C. albicans* [129], *C. parapsilosis* [130], and *C. auris* [131,132]. While *C. auris* is usually treated successfully with echinocandins, some clinical isolates are not only multidrug resistant but also pan-resistant to all antifungal drug classes used in the clinic [103,133].

The WHO has determined antimicrobial resistance to be among the top ten global public health threats. While it affects the health of individuals, increased limitations on the ability to carry out many standard surgical procedures means antifungal resistance has an increasingly significant economic cost. The prevention and treatment of fungal infections now often requires prophylaxis, longer hospital stays and more expensive medicines [55].

The fungistatic activity of azoles and the poor bioavailability of echinocandins have led to the consideration of novel therapeutic options. The simultaneous administration of two or more antifungal agents aims to achieve several therapeutic goals, including (i) the prevention of drug resistance by inhibiting multiple fungal targets, (ii) the minimization of side effects and toxicity associated with high doses of single agents by synergistic reductions in drug dosage, and (iii) the treatment of challenging resistant infections caused by multidrug resistant pathogens.

The repurposing of existing drugs in drug combination is an important evolving strategy in antifungal therapy, especially in combating *Candida* species. Considerable research has identified synergistic effects between existing antifungal agents with different modes of action, e.g., azoles in combination with echinocandins [134], polyenes [135], or inhibitors of drug efflux [27,136]. These studies show enhanced efficacy can be delivered by targeting multiple cellular components or pathways in fungi.

**Figure 2:**
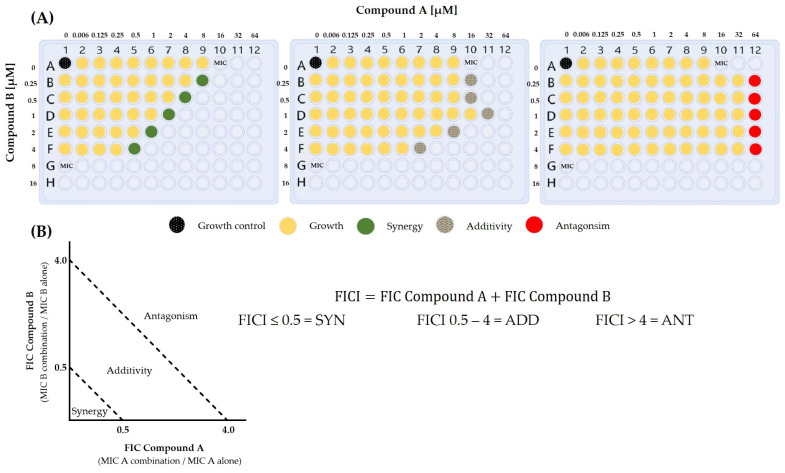
Visualization of the effects achievable by combination therapy. (**A**) Theoretical microtiter plates showing synergy, additivity, and antagonism. Synergy (SYN) occurs when the combination of two compounds enhances each other’s effects, thereby increasing, for example, the inhibition of fungal growth. Antagonism (ANT) is observed when the effects of two compounds together result in a worse outcome than either compound alone. Additivity (ADD) occurs when the combined effect of both compounds is no greater than the effect of each compound individually. (**B**) Theoretical diagram showing synergy assessment. FIC = fractional inhibitory concentration, FICI = FIC indices.

Interactions between compounds lead to biological effects described using the terms synergy, antagonism, and no interaction (Figure 2) [137]. However, the definition of “interaction” is debated [138,139]. Synergy or antagonism are usually defined as a greater or worse effect of the combined drugs, respectively, compared with the single effect of the individual drugs, whereas the term no interaction (also referred as additivity or additive effect) has various interpretations [138,140,141,142]. 

Some interactions that underpin drug synergy can be mimicked in the deletion mutants of haploid *S. cerevisiae*. In this scenario, the deletion of one gene is not lethal but the deletion of a second gene is. For example, the deletion of a gene affecting drug efflux such as the *PDR3* transcriptional regulator, the *PDR5* ABC transporter, or the *MDR1* MFS drug efflux pump genes is non-deleterious, but it confers increased sensitivity or even lethality in response to a transported drug (i.e., azole drugs) that inhibits the protein product of the second gene, such as Erg11. This approach may be more broadly applicable to antifungal substrates of drug efflux that target intracellular elements of essential fungal gene products including the plasma membrane proton pump, glucan synthase, chitin synthase, etc. However, it is important to realize that azoles may respond differentially to the activity of drug efflux pumps. For example, all azole drugs in clinical use are substrates of drug efflux mediated by the dominant ABC transporters such as Pdr5 in *S. cerevisiae* and Cdr1 in *C. albicans* [143,144]. While short- and medium length-tailed azoles such as FLC, VCZ, and ISA are substrates of the MFS efflux pump CaMdr1, the long-tailed azoles ITC and POS are poor substrates of these transporters. Furthermore, the echinocandins are not chemosensitized by efflux pump inhibitors as echinocandins are not substrates of drug efflux [53].

### 3.1. Combination Therapies Used in the Clinic

Combination therapy has numerous clinical applications. Developed in response to the emergence of penicillin resistance, Amoxacillin (penicillin plus clavulanic acid) provides a therapeutic combination still widely used to treat bacterial infections. Combinations of several drugs are frequently used in cancer chemotherapy (reviewed in [145]). Viral infections, such as HIV/AIDS [146] or Coronavirus disease (COVID-19) [147], are often treated with dual or even triple therapies. For fungal pathogens, the recommended induction phase of cryptococcal meningitis treatment uses AMB plus Flucytosine [148,149], with AMB plus FLC used when expense precludes Flucytosine. FLC is then used in consolidation and maintenance therapies for up to the year required for fungal clearance [150]. Apart from that example, monotherapy is still the gold standard to treat fungal infections. However, some clinical studies have evaluated the effects of combination therapy on patients suffering from severe fungal infection. For example, two independent studies evaluated the combination of VRC and CAS or VRC and ANA against invasive aspergillosis. The results showed that the combination VRC + CAS reduced the mortality rate and increased the three-month survival rate compared with treatment with VRC alone [151], and VRC + ANA led to higher survival rates than monotherapy [152].

Another study investigated the effect of combination therapy against invasive mucormycosis caused by *Lichtheimia corymbifera*, *Mucor* spp., *Rhizopus* spp., *Rhizomucor* spp., or *Cunninghamella bertholletiae*. A clinical improvement was shown for 56 % of the patients when liposomal AMB was combined with POS [153].

The current use of antifungal combination therapy against *Candida* has been summarized in several reviews [25,28,141,154], including against *C. auris* [155,156]. The following is a brief overview of the literature describing combinations that target multiple pathways or individual cellular components in *Candida* and related genera, with a focus on *C. albicans* and especially *C. auris*. 

### 3.2. Combination Therapies in Development

- Synergistic combinations affecting the efflux of azole drugs

In *Candida* spp., two major groups of efflux pumps are involved in the conferral of antifungal resistance, namely major facilitator superfamily (MFS) and ATP-binding cassette (ABC)-type transporters [157]. The activity of both pumps results in the net transport of a wide array of substances across the fungal cell membrane. Their drug efflux can be assayed using fluorescent substrates such as Nile Red or rhodamine 6G (R6G), or by measuring the ATPase activity of ABC transporters where ATP hydrolysis completes the drug transport reaction cycle. 

ABC transporters are found in all organisms, with most of them residing in the plasma membrane. They are primary transporters that use energy from ATP hydrolysis to transport macromolecules or small molecules against a concentration gradient [157,158,159]. Most ABC proteins in fungi are large (~140 kDa) and are considered full-size ABC transporters of the type [NBD-TMD]_2_ [33]. They consist of two hydrophilic nucleotide binding domains located in the cytoplasm and two hydrophobic transmembrane domains (NBDs) containing multiple (usually 6) α-helical transmembrane segments (TMSs) [160,161,162]. There are also half-size transporters with one NBD and one TMD that likely operate as homodimers or heterodimers [160]. The NBD pairs contain protein motifs conserved among eukaryotes [157,163]. Of importance for nucleotide binding are the Walker A motif, also known as phosphate-binding loop (P-loop, G-X-X-G-X-G-X-S/T) [164], and the Walker B motif (h-h-h-h-D, “h” stands for hydrophobic residue) [164]. The highly conserved ABC signature motif or C-loop (L-S-G-G-Q) is present in all ABC transporters [165]. The use of the fungal NBDs as drug targets is likely to be nonspecific and hence toxic to human cells. Targeting the TMDs of fungal ABC transporters may provide better opportunity for antifungal specificity.

MFS pumps are secondary transporters (typical length 400–600 amino acids, <70 kDa) that translocate small molecules powered using an electrochemical transmembrane gradient [166,167,168]. Like ABC proteins, most MFS pumps have two TMDs and consist of 12 or 14 TMSs [169,170]. Mdr1 is the best characterized member of the *C. albicans* DHA1 family and appears to play an important role in clinical resistance to azoles [171]. Furthermore, Wirsching et al. [172] observed a strong link between *MDR1* overexpression and FLC resistance in clinical isolates. 

The functional overexpression of efflux pumps such the Cdr1 or Mdr1 in a hypersensitive *S. cerevisiae* host confer pleiotropic drug resistance to a wide range of drug efflux substrates. Like CaCdr1, CauCdr1 overexpression confers strong resistance to all azole antifungal drugs used in the clinic [173]. And like CaMdr1, CauMdr1 overexpression confers strong resistance to a subset of the azole drugs and much weaker resistance to the long-tailed azoles ITC and POS [173]. Experiment systems using drug efflux pump overexpression to confer azole resistance have enabled the discovery of specific inhibitors of fungal drug efflux pumps. 

The precursor of the D-decapeptide derivative RC21v3 was discovered by screening a 1.8-million-member D-octapeptide combinatorial library using overexpressed CaCdr1 as a target and overexpressed CaMdr1 as a counter screen in a *S. cerevisiae* host strain deleted of drug efflux pumps [174]. After optimization, RC21v3 was found to be a highly specific inhibitor of CaCdr1 (it did not inhibit the activity of CgCdr1), and its acting on the extracellular surface of this efflux pump by selecting and characterizing suppressor mutants that were susceptible to azole drugs. The application of RC21v3 at 0.02 µM potentiated the activity of several azole drugs and was shown to be effective in a murine model of oral infection by an azole resistant strain of *C. albicans* [175]. The combination of azole and RC21v3 reduced the number of colony-forming units sampled from the oral cavity and reduced oral lesions compared with azole alone. 

A similar strategy using overexpressed CaMdr1 as a target and CaCdr1 as a counter screen identified the squarile compound (3-(phenylamino)-4-{(1,3,3-trimethyl-2,3-dihydro-1H-indol-2-ylidene)methyl}cyclobut-3-ene-1,2-dione) as a specific inhibitor for CaMdr1 [136]. Both RC21v3 and the squarile compound blocked the drug efflux of their respective targets, e.g., ATPase activity and glucose-dependent R6G efflux by CaCdr1, and glucose-dependent Nile Red efflux by CaMdr1. Checkerboard assays confirmed the synergistic effect of FLC and the squarile compound with FICI values < 0.05.

Yong et al. [176] investigated the effects of berberine hydrochloride (BBH), a compound used in traditional Chinese medicine. A combination of BBH and FLC significantly decreased (32–512-fold) the MIC of FLC for a biofilm forming FLC-resistant strain of *C. albicans*. The combination decreased drug efflux through berberine inhibition of CaCdr1 activity measured as R6G efflux. *CDR1* mRNA expression levels were down regulated by FLC + BBH treatment whilst FLC alone increased *CDR1* expression levels. The combination also reduced adhesion and biofilm formation which the authors explained as the possible downregulation of hyphal-specific genes *HWP1* and *ALS3*. They also highlighted the effects of the BBH/FLC combination on vacuolar calcium regulation genes that enabled increased cytoplasmic Ca^2+^ levels. However, the molecular basis of BBH’s mode of action in increasing susceptibility to azole drugs has yet to be determined. 

Clorgyline was found to be an inhibitor of both CaCdr1- and CaMdr1-dependent drug efflux in the yeast expression system and in some *C. albicans* clinical isolates that overexpress these efflux pumps [177]. We subsequently identified the potential of Clorgyline derivatives M19 and M25 to combat azoles resistance in *C. auris* [178]. Both compounds act synergistically with azoles, especially POS (FICI ≤ 0.3) against azole resistant and azole susceptible clinical isolates of Clade I and II, respectively. Moreover, M25 negates azole resistance by inhibiting both ABC (CauCdr1) and MFS (CauMdr1) classes of efflux pump transporters. Whether Clorgyline and its analogues directly bind to the efflux pumps or affect the lipid environment of these molecules has yet to be determined.

The antibiotic Gentamicin strengthened the antifungal activity of azoles against *C. albicans*, with FICI values of 0.13–0.14. Suppression of efflux pump activity, detected as reduced R6G efflux and inhibition of extracellular phospholipase activity, was found to be responsible [179]. However, the use of Gentamicin in pregnant women and children is not recommended due to short term effects on kidney and neuromuscular function and long-term side effects such as irreversible bilateral congenital deafness.

- Inhibitors of transcriptional regulators of drug efflux

Thakur et al. [180] investigated the transcriptional regulation of the *S. cerevisiae* Pdr5 efflux pump by considering interaction between the Pdr1 transcriptional regulator and the KIX component of the transcription complex. Efflux pumps such as S. *cerevisiae* Pdr5 or *N. glabratus* Pdr5 can be induced by xenobiotics like Ketoconazole. Due to similarities between the mammalian nuclear Pregnane X Receptor (PXR) and fungal Pdr1/Pdr3, it was expected that interactions of xenobiotics with Pdr1 or Pdr3, respectively, might stimulate the expression of target genes. Human PXR plays a crucial role in regulating the expression of multidrug resistance (MDR) genes, such as the drug efflux transporter P-glycoprotein, in response to xenobiotics [181,182]. The group confirmed the importance of the Gal11 subunit in the Mediator co-activator complex for Pdr1/Pdr3-dependent transcriptional activation of target genes involved in MDR. The deletion of *GAL11* strongly diminished the xenobiotic-induced expression of MDR genes like *PDR5*. The Gal11 KIX domain physically interacts with the activation domain of Pdr1, an interaction enhanced by the presence of xenobiotics [180]. The structure of the Gal11 KIX domain is very similar to the KIX domains of human Mediator (Med) 15, and it also binds to the Pdr1 activation domain [183]. Mutational analysis revealed molecular details of this interaction by identifying key residues in the Gal11 KIX domain important for binding to the Pdr1 activation domain. The exogenous expression of wild-type Gal11, but not KIX domain-deleted Gal11, rescued the xenobiotic sensitivity and defects in MDR gene expression observed in the *GAL11* deletion strain. This demonstrated the functional importance of the Gal11 KIX domain in Pdr1/Pdr3-mediated xenobiotic response and MDR. It was concluded that the Gal11 KIX domain, which provides the Pdr1 activation domain docking site, might act as a promising drug target for the future. Additional experiments are needed to assess such potential benefits. This study provides a basis to discover potential KIX domain inhibitors. Newly designed inhibitors, such as peptides [184], can be tested in vitro in combination with antifungals to evaluate their synergistic effects. However, similarity to human PXR may make unspecific binding and unwanted side effects problematic.

- Synergy mediated by inhibitors of Pma1p

The H^+^-ATPase Pma1 is an essential enzyme responsible for maintaining the proton gradient across the plasma membrane in fungi. It plays a crucial role in fungal cell viability by regulating the pH and ion homeostasis. The essential nature of Pma1 in fungi and features of this P-type ATPase specific to fungi makes it an attractive target for antifungal therapy without affecting human cells [185]. Pma1p was confirmed to be a druggable antifungal target by showing that the proton pump inhibitor (PPI) acid-activated Omeprazole (OME) inhibits Pma1 at the cell surface [186,187]. That work prompted the testing of OME in combination with POS, VRC, and ISA against *S. cerevisiae* strains overexpressing CauCdr1 and CauMdr1 [178]. Checkerboard assays revealed that the combination of OME with POS and VRC acted synergistically against strains overexpressing CauCdr1 and CauMdr1, whereas ISA + OME synergized only against strains overexpressing CauMdr1. It was suggested that the inhibition of Pma1 by OME depleted the plasma membrane electrochemical gradient, which reduced CauMdr1 activity. It was speculated that the reduced activity against strains overexpressing CauCdr1 was due to a competition between the azoles and the proton pump inhibitor at the drug-binding site of CauCdr1. Tests with Rabeprazole, a PPI activated at higher pH than OME, showed synergy with POS but not VRC against strains overexpressing Mdr1 but not Cdr1. 

Monk et al. [188] screened a 1.8-million-member D-octapeptide combinatorial library and tested for the inhibition of *S. cerevisiae* and *C. albicans* growth with Pma1 as the main drug target. Optimization by N-terminal extension identified the D-decapeptide BM2 as a potential broad-spectrum inhibitor targeting Pma1. BM2 showed fungistatic (≤5 µM) or fungicidal (≤10 µM) activity against *Candida* spp., *Cryptococcus neoformans*, and *S. cerevisiae* by inhibiting the physiological activity of Pma1. In Pdr5 overexpressing *S. cerevisiae* strains, BM2 inhibited ATPase activity and R6G efflux in a dose-dependent manner. BM2 at sub-MIC levels (≤2.5 µM) chemosensitized recombinant *S. cerevisiae* strains overexpressing various ABC or MFS efflux pumps, or Erg11 to FLC. Additionally, BM2 (<10 µM) chemosensitized in some FLC-resistant clinical isolates of *N. glabratus*, *C. albicans*, and *Candida dubliniensis* to FLC and chemosensitized a *P. kudriavzevii* clinical isolate to ITC determined by checkerboard drug chemosensitization assays. A limitation of D-peptides such as BM2 or RC21v3 is that their production is expensive and technically challenging. Additionally, because minor toxicity was observed in cultured Hep2 cells, the use of D-decapeptides might be more suitable for superficial rather than invasive fungal infections.

- Synergy of azoles with inhibitors of glucan synthase

Echinocandins target the β-1,3-glucan synthase located in the fungal plasma membrane while azoles target the lanosterol 14α-demethylase in the endoplasmic reticulum. By targeting separate biosynthetic pathways, echinocandin plus azole combinations should be at least additively effective against a wide range of *Candida* spp. and help to prevent the development of antifungal drug resistance. 

Fakhim et al. [189] tested combinations of the echinocandins CAS and MFG with the azoles FLC and VRC against clinical isolates of *C. auris,* including strains resistant to FLC and MFG. Only the combination of MFG with VRC gave synergy, with FICI values between 0.15 and 0.5. Caballero et al. [21] found ISA synergized with CAS, ANA, or MFG and stopped fungal growth for 48 h in time-kill experiments. 

Pfaller et al. [190] tested ANA combinations with ISA or VRC against a collection of *C. auris* echinocandin susceptible clinical isolates using the checkerboard technique. The ISA plus ANA combination gave a synergy for ~31% of the isolates, while ISA + VRC gave synergy for only ~14% of the isolates. 

Further research is needed to evaluate the individual and collective impacts of azoles and echinocandins on the enzyme activities of β-1,3-glucan synthase and lanosterol 14α-demethylase among the various clades of *C. auris*, as well as the effects of glucan synthase inhibition on the access of azoles to the endoplasmic reticulum. 

While the combination of azoles and echinocandins can be beneficial in certain fungal infections (particularly resistant fungal infections), there is not enough evidence to support the use of this combination for all fungal infections. Clinical benefits may depend on the fungus causing the infection and the patient’s condition.

- Single molecule dual target inhibitors

Some recent studies have focused on identifying compounds that are dual-target inhibitors of enzymes responsible for drug resistance. 

Sun et al. [191] designed novel inhibitors that simultaneously target fungal Cyp51 and the programmed death-1 (PD-1) ligand (PD-L1) in the human host. The human PD-L1 protein binds to T-cells that express PD-1. The binding of PD-L1 to PD-1 stops T-cells from killing cells that express PD-1 [192]. In cancer therapy, inhibitors that block PD-L1 binding to T-cells enable immune cell attack on cancer cells [193]. Sun et al. [191] aimed to enhance the therapeutic efficacy of the fungistatic azole drugs and thereby reduce the emergence of drug-resistant fungal strains. By analyzing the structural scaffolds of Cyp51 inhibitors and PD-1/PD-L1 inhibitors, they designed novel compounds that could bind to both targets. The skeleton growth method employed to construct novel quinazoline derivatives allowed the flexible matching of molecular structures with complex active regions, ensuring optimal binding properties. The compounds tested exhibited antifungal activity against various *Candida* spp., with MIC_50_ values ranging from 0.25 to 2.0 mg/L. The compounds inhibited Cyp51 activity, and induced ROS production and mitochondrial damage, ultimately leading to fungal lysis and death. Blocking interaction between PD-L1 and PD-1 enhanced the host immune response, accelerating recovery from fungal infection. Evaluation in vitro and in vivo demonstrated the effective inhibition of fungal proliferation and the faster recovery of infected tissues.

Liu et al. [194] also analyzed bifonazole derivatives as potential dual-target enzyme inhibitors. The compound 14a-2 had broad spectrum activity against *Candida* spp. including *C. albicans*, with MIC_50_ ranging from 0.5 to 0.125 mg/L. The compound was suggested as a potential candidate for drug development due to a high affinity to the Cyp51 active site (IC_50_ = 0.17 µM) like the control drug Ketoconazole, and an activity against PD-1 comparable to the positive control. 

Cyp51/PD-L1 inhibitors were also designed using a fragment-based approach [195]. Tests with various fungi, including *Candida* spp., identified 18b-2 as the best hit. MIC_50_ values ranged from 0.125 to 0.2 mg/L against yeast and the mold *A. fumigatus*. Compound 18b-2 exhibited a stable binding affinity to both targets. 

Zhou et al. [196] designed the compound (Gly_0.8_Nap_0.2_)_20_ to target fungal DNA and the membranes of various multidrug resistant fungi including *Candida* spp. A mouse model found promising in vivo activity against multidrug resistant *C. albicans*.

- Animal trials of combination treatments

Few in vivo studies have investigated the effects of azoles in combination with non-antifungals. Selected examples are discussed below and summarized in Table 2. 

The inhibitory effect of the antibiotic sulfamethoxazole (SUL) was investigated by Eldesouky et al. [197]. SUL interferes with the folic acid synthesis in bacteria. The combination with trimethoprim inhibits nucleic acid biosynthesis and bacterial growth [198]. The combination of SUL with VRC or ITC, but not FLC, restored susceptibility in azole-resistant *C. auris* strains from Clade I and III. In vivo experiments with *Caenorhabditis elegans* confirmed this effect by showing a nearly 70% enhancement of survival rate with SUL + ITC. The synergy appears to involve interference with the fungal folate pathway, which is linked to ergosterol biosynthesis via NADPH-cytochrome P450 reductase which contains both FMN and FAD cofactors. Strains that are azole resistant due to overproduction or decreased affinity for CauErg1 were highly susceptible to the SUL–azole combination. However, the combination was not synergistic for strains showing azole resistance via efflux pump hyperactivity. This study underscores the potential of the fungal folate biosynthetic pathway as a target to enhance the activity of azole antifungals in treating infections caused by *C. auris* Clade I strains. 

The combination with azoles of the HIV protease inhibitors Lopinavir (LPV) and Ritonavir (RTV) was evaluated in two separate studies [199,200]. LPV or RTV alone had limited effectiveness against *C. auris*. In contrast, the combination of LPV with FLC, VRC, or ITC showed strong synergy against the yeast, with LPV plus ITC (FICI ≤ 0.31) being most potent. In addition to in vitro findings, combinations of LPV or RTV with azoles significantly reduced fungal burden in the kidneys of infected mice, with LPV-Ritonavir (LPV-R) plus ITC being particularly effective. Notably, the protease inhibitors were used at clinically achievable concentrations. The molecular basis of the synergy of LPV with azoles has yet to be determined.

In vitro studies showed that the TOR kinase inhibitor AZD8055 did not exhibit significant antifungal activity, but when combined with azoles it demonstrated synergistic effects against *C. albicans*, *C. auris*, *A. fumigatus*, and *C. neoformans* (FICI ≤ 0.5) [201]. Synergy was most frequently observed with POS, particularly against azole-resistant *C. auris*. In some cases, this combination reduced azole MICs by up to 16-fold. In vivo experiments using the larval model *Galleria mellonella* confirmed the improved antifungal activity due to the AZD8055 + ITC combination, resulting in enhanced survival rates compared with azoles alone. The study suggests that AZD8055-azole combination potentiates antifungal activity and may help overcome azole resistance. AZD8055 is an orally bioavailable, potent, and specific inhibitor that binds to the ATP binding cleft of TOR kinase and inhibits both TORC1 and TORC2 [202]. AZD8055 was initially developed for cancer therapy and may serve as a promising adjunct to antifungal treatments for cancer patients at risk of fungal infection. 

Eldesouky et al. [203] discussed the potential of the antiemetic drug Aprepitant, a neurokinin-1 antagonist, as a novel azole chemosensitizer of multidrug-resistant *C. auris* and other *Candida* spp. Aprepitant enhances the antifungal activity of azole drugs, particularly ITC, in both in vitro and in vivo experiments. Aprepitant plus ITC showed broad-spectrum antifungal activity and converted ITC from a fungistatic to a fungicidal agent against *C. auris*. The authors suggested their results reflected interference with iron homeostasis in *C. auris*. Eldesouky et al. [204] tested the selective estrogen receptor modulator Ospemifene in growth kinetic and checkerboard assays to show synergy with ITC against intrinsically azole resistant *C. auris* clade I and IV strains. This effect was confirmed in the *C. elegans* model in which the fungal burden was reduced by >90%. The authors suggested the synergy was most likely elicited through direct interference with *C. auris* efflux pumps. However, other azoles such as FLC or VRC failed to synergize with Ospemifene. 

The therapeutic potential of the D-decapeptide derivative RC21v3 (0.02 µmol per dose) was investigated in a murine oral infection model caused by azole-resistant and azole-susceptible *C. albicans* [175]. In mice infected with azole-resistant *C. albicans*, treatment with either FLC (0.3 mg/kg of body weight per dose) or ITC (0.16 mg/kg of body weight per dose) was only partially successful. However, the combination with RC21v3 at 0.3 mg/kg body weight per dose reduced the oral infection burden (determined by CFU counting) and diminished the lesion score caused by *C. albicans* to almost zero, which was confirmed by histological examination.

**Table 2:** Drug combinations of azoles and inhibitors tested in vivo against *C. auris*.

Azole	Combination	Drug Information	Result	Setting	Source
FLC, VRC, ITC	Sulfamethoxazole	Antibiotic, CYPC9 inhibitor	Active against Erg11, ineffective against efflux pump overexpression; FLC limited effect (SYN one strain only); SUL+VRC or ITC: SYN or ADD in vivo: VRC + SUL 70% survival rate after 5 d	in vitro, *C. elegans* model	[197]
FLC, VRC, ITC	Lopinavir	HIV protease inhibitor, CYP3A substrate	LPV+FLC or VRC: SYN or ADD; LPV+ITC: SYN in vivo: LPV+ITC reduced burden	in vitro, *C. elegans* model	[199]
FLC, VRC, ITC	Lopinavir, Ritonavir		RTV interfered with efflux pump; RTV+FLC, VRC, or ITC: SYN or ADD; LPV+FLC or VRC: SYN or ADD; LPV+ITC: SYN in vivo: LPV+RTV+FLC or ITC reduced burden in kidneys	in vitro, mouse model	[200]
VRC, ITC, POS	AZD8055	ATP-competitive inhibitor of mTOR kinase activity; controller of cell growth and proliferation in eukaryotes	AZD8055+VRC, ITC, or POS: SYN or ADD in vivo: ITC+AZD8055 better than ITC alone	in vitro, *G. mellonella* model	[201]
FLC, VRC, ITC	Aprepitant	Antiemeticum, neurokinin-1 antagonist, dose-dependent inhibitor and inducer of CYP3A4	APR+FLC, VRC, or ITC: SYN or ADD; APR+ITC active against biofilm in vivo: APR+ITC fungicidal in time killing assay *C. elegans:* APR+ITC reduced burden	in vitro, biofilm, *C. elegans* model	[203]
ITC	Ospemifene	Selective estrogen receptor modulator	OSP+ITC: SYN	in vitro, *C. elegans* model	[204]
FLC, ITC	RC21v3	D-decapeptide Derivative	Combination reduced oral lesions and CFU count	Oral murine infection model	[175]

Abbreviation: SYN = synergy; ADD = additivity

- Combinations in clinical practice

Combining compounds aims to reduce the opportunity for acquired resistance development and may confer long-term advantage by protecting the medical and economic value of existing antifungals. Unfortunately, the worldwide usage of antifungals as pesticides is increasing and most agrochemical azoles have structures like azole drugs used in medicine [205,206]. The dual use of azoles, particularly their widespread use in agriculture, has accelerated the development of resistance to this important class of antifungals. As has been documented for *A. fumigatus* CYP51A mutations that confer resistance to VRC in azole naïve patients [207,208,209], this problem is already limiting therapeutic options.

Solving this problem by discovering and developing novel antifungals is a crucial but time-consuming and expensive task. And while sensitizers that increase antifungal efficacy are eagerly sought, implementing combination therapy in clinical practice has significant challenges. Patients will need to be closely monitored as combining medications may increase the risk of adverse effects due to drug interactions, which can lead to unpredictable effects including drug toxicity and reduced efficacy. When multiple drugs are used together, risks can arise due to the interactions between the drugs. For example, one drug may inhibit the metabolism of the other drug, leading to increased and/or prolonged drug exposure. Or the risk of organ damage may increase if both drugs have similar toxicity on the same organ. Potential interactions need to be evaluated systematically and, where applicable, more suitable, dose adjusted, formulations need to be prepared for drug delivery.

It can be difficult to determine which drug is causing toxicity. This makes monitoring complex, as side effect may be attributed incorrectly. Regulatory compliance imposes additional requirements and costs, with the need for components to be approved both individually and as combinations. This economic problem highlights the value of repurposing approved drugs when seeking chemosensitizers (discussed in Holmes et al. [27]). Examples of that approach include Clorgyline, FK506, and their analogs [177,178,210]. 

Therapy combining compounds that synergistically target different cellular pathways should decrease the likelihood of drug resistance development. However, in certain infectious diseases, combination therapy can promote the development of drug resistance due to drug regime failure [211,212].

Despite theoretical advantage supported by laboratory experiments, robust clinical evidence establishing the value of combination therapy over monotherapy is usually lacking. However, some combinations do successfully treat fungal infections. For example, the recommended treatment of cryptococcal meningitis is a combination of AMB with Flucytosine or FLC. This combination reduces monitoring requirements and mortality rates, and improves cerebrospinal fluid clearance compared with monotherapy [213]. The Infectious Diseases Society of America suggests the same combination (AMB + Flucytosine) for the initial treatment of central nervous system infections or valve endocarditis due to *Candida*, and for azole-resistant *N. glabratus* infections [19]. 

Greater insight into synergy between conventional antifungals such as the azoles or echinocandins with novel compounds or repurposed drugs is needed to identify conditions that maximize susceptibility and minimize opportunity for resistance and side effects. This requires an understanding of (a) the mode of action of individual antifungal drugs at the level of target-ligand structures (e.g., high resolution Cyp51-azole and Fks1-echinocandin structures), (b) knowledge of the molecular mechanisms that underpin synergistic interactions and their physiological consequences (e.g., increased intracellular azole concentrations due to reduced drug efflux, interactions with key host enzymes responsible for possible side effects), and (c) the many causes of acquired drug resistance at the atomic and physiological levels (e.g., drug target overexpression, resistance due to drug target mutations). An important way forward will be to obtain high resolution crystal structures of representative Cdr1 and Mdr1 drug efflux pumps from *Candida* species. For example, the cryo-EM structures of *C. albicans* Cdr1 apo-enzyme, with its substrate FLC and with the inhibitor milbemycin-oxime, have recently been obtained [214]. This information will help in the design of novel drugs that are not drug efflux pump substrates and identify new targets for drug development. 

## 4. Conclusions

Antifungal combination therapy is a promising strategy to address the growing challenges of antifungal resistance and limited treatment options. The incidence of resistant fungal infections, particularly among *Candida* spp. such as the emerging pathogen *C. auris*, makes the need for more effective therapeutic approaches critical. Combining antifungal agents can offer several practical advantages, including the prevention of resistance development by targeting multiple pathways, reduced drug dosage, and minimized toxicity, and the treatment of infections caused by multidrug-resistant pathogens.

This review highlights combinations that showed promising results in vitro, such as the Clorgyline derivatives M19 and M25, or berberine hydrochloride, which can be considered for further in vivo studies before advancing to clinical trials. The review also provides an overview of substances already in clinical use (e.g., Aprepitant) which can be considered for antifungal combination therapy in clinical trials. However, implementing combination therapy in clinical practice presents challenges such as potential drug interactions, an increased risk of adverse effects, as well as the possibility of promoting drug resistance in certain cases. Despite these challenges, research into antifungal combination therapy has progressed by demonstrating synergistic effects between different classes of antifungal agents. Understanding the molecular mechanisms that underpin such synergistic interactions and drug resistance will help with the design of more effective treatment strategies and novel antifungal drugs. This may include individual drugs able to inhibit multiple targets.

The multiple threats posed by antifungal resistance in *Candida* species, a limited resource of antifungal agents, and the emergence of new fungal pathogens driven by medical practices and changing ecology underscore the need to further explore combination therapy to obtain better treatment outcomes. While numerous combinations have been explored for their in vitro efficacy, further research is needed, especially with animal models and in clinical trials, to validate the efficacy and safety of combination regimens, ultimately paving the way for more robust and comprehensive approaches to antifungal treatment of value to medicine.

## Figures and Tables

**Table 1:** Overview of novel antifungals in the drug discovery pipeline.

Antifungal Class	Antifungal	Mode of Action	Information	Source
N-phosphonooxymethyl manogepix prodrug	Fosmanogepix (APX001)	The active form manogepix targets the fungal enzyme Gwt1. Cell wall integrity is impaired and fungal growth inhibited	Active against *Candida* except *P. kudriavzevii*	[106,107,108]
Triterpenoid	Ibrexafungerp (SCY-078, MK-3118)	Inhibitor of β-(1,3)-d-glucan synthase, like the echinocandins, but with different enzyme binding site	Active against *Candida* including *C. auris* and *N. glabratus*	[109,110,111,112,113,114,115]
Echinocandin	Rezafungin (CD101)	Inhibitor of β-(1,3)-d-glucan synthase	Active against *Candida* including *C. auris*	[116,117,118,119]
Tetrazoles	Oteseconazole (VT-1161), VT-1598 *, VT-11134 *, VT-1129 ^†^	Disruption of the sterol biosynthetic pathway by inhibition of CYP51	Active against *Candida* including *C. auris*, and FLC and echinocandin resistant *N. glabratus*	[120,121,122,123]

* Phase 1 Clinical Trial, ^†^ Investigational.

## Data Availability

No new data were created or analyzed in this study. Data sharing is not applicable to this article.
